# 

**Published:** 1886-10-16

**Authors:** 


### THE HOSPITAL

An Institution, Family, and Parochial Journal of
Hospitals, Asylums, & all Agencies for the Care of the Sick, Criticism & News.

### CONTENTS.

Ought Asylums to Rank as Hospitals? - - - 35

Discharged. Not where to Lay his Head. By George Fleming. Chapter IV - - - - - 36

The Hospital Problem.—The Utility and Necessity of Hospitals.—The Facts stated.—Few Voluntary Supporters. By the Rt. Hon. the Earl of Derby - 38

The Hospitals of our Grandfathers, at Home and Abroad - - - - - - - - 39

Words of Consolation - - - - - - - - 40

Hospital Administration.—Alcohol at the London Hospital - - - - - - - - 41

Notes and News.—H.R.H. the Prince of Wales and the Exhibition Boxes.—Prince Albert Victor at Burnley.—Successful Architects.—Dr. Hazlerigg and Devonshire Hospital, Buxton.—The Earl of Derby at Manchester.—Staff-nurses promoted.—Bath Hospital, Harrogate.—Resignation of Mr. Worthington.—Devonshire Hospital, Buxton.—An Accident.—Amalgamation in Sunderland.—Improvements at Westminster Hospital, etc. - 42

The Groan of the Willing Horse - - - - - 45

Annotations.—Dangerous Reticence.—Hospitals and Rates.—Hope for the Hopeless - - - - - - 45

Flowers, Ferns, and Ward Decorations.—The Charm and Beauty of Flowers.—Christ and the Flowers - - 47

Editor's Letter Box.—Officers' Food and the Regulation of Meals.—"The Hospital," Elementary Schools and the Public - - - - - - - - 48

Prize Competitions - - - - - - - - 49

The Hospital Charity Box. Prizes and Results - - 49

The Children's Corner. Puzzles, Answers, and Correspondence - - - - - - - - 50

Notices to Correspondents, etc., etc. - - - - 50

Help at Hand, Brother.—Convalescents' Employment and free Advertisement columns - - - - - v

